# The Neurogenic Compound P7C3 Regulates the Aerobic Glycolysis by Targeting Phosphoglycerate Kinase 1 in Glioma

**DOI:** 10.3389/fonc.2021.644492

**Published:** 2021-06-18

**Authors:** Wenjin Chen, Weiqiang Jia, Cuiying Wu, Lihua Chen, Kai Sun, Ji Wang, Boyun Ding, Ning Liu, Ruxiang Xu

**Affiliations:** ^1^ The Second School of Clinical Medicine, Southern Medical University, Guangzhou, China; ^2^ Department of Neurosurgery, The Seventh Medical Centre, Chinese PLA General Hospital, Beijing, China; ^3^ Department of Neurosurgery, Sichuan Academy of Medical Science and Sichuan Provincial People’s Hospital, University of Electronic Science and Technology of China, Chengdu, China

**Keywords:** P7C3, human proteome microarray, aerobic glycolysis, PGK1, glioma

## Abstract

**Background:**

P7C3 is a neurogenic compound that exhibits neuroprotective properties in neural cells. However, its target proteins and effects in glioma are unknown.

**Methods:**

The candidate P7C3 target proteins were analyzed using a human protein microarray containing 23136 human proteins. A streptavidin agarose affinity assay was used to verify the direct interaction between P7C3 and phosphoglycerate kinase 1 (PGK1). Mass spectrometry was used to identify the binding sites of PGK1 for P7C3 binding. Seahorse XF96 extracellular flux analyzer was used to measure the cell oxygen consumption rate and extracellular acidification rate. Glycolytic metabolites were measured using the related kits. Protein level was detected by western blotting and immunohistochemical staining. Autophagy was analyzed using a transmission electron microscope and western blotting. The malignancy of tumor progression *in vitro* and *in vivo* was analyzed based on cell viability, apoptosis and proliferation, migration and invasion, and xenograft model. Glial cells were marked by antibodies *via* immunohistochemical staining.

**Results:**

The human protein microarray identified 577 candidate P7C3 target proteins. The global profile of P7C3 target proteins indicated that P7C3 regulates glycolysis. Metabolic experiments confirmed that P7C3 regulates aerobic glycolysis in glioma cells. The underlying mechanism of P7C3 was found to be direct targeting PGK1 at lysine residues and asparagine residues, and the specific P7C3-PGK1 interaction led to decreased protein level and total intracellular kinase activity of PGK1. The Cancer Genome Atlas and Chinese Glioma Genome Atlas databases indicated that the mRNA level of PGK1 is significantly increased in high-grade glioma, and the abnormally high mRNA level of PGK1 is associated with a poor prognosis in patients with glioma, suggesting that PGK1 is a promising target for glioma therapy. The inhibition of PGK1 and the subsequent suppression of aerobic glycolysis caused by P7C3 inhibited the malignant growth of glioma *in vitro* and *in vivo*. Furthermore, P7C3 did not damage normal glial cells under concentration, which exhibit an inhibitory effect on gliomas.

**Conclusions:**

This study revealed that P7C3 suppresses glioma by regulating aerobic glycolysis *via* directly targeting PGK1. Furthermore, we identified the P7C3 target proteins for the first time which is expected to provide scientific clues for future studies.

## Introduction

P7C3 was firstly reported as a compound that exerts pro-neurogenic activity by protecting newborn neurons and enhancing neurogenesis in adult mice (1). Follow-up studies showed that a series of improved active derivatives of P7C3, such as P7C3-A20 and P7C3-S243, further strengthen its pharmacological functions (2, 3). These active derivatives of P7C3, P7C3-A20 exhibited neuroprotective activity in an animal model of Parkinson’s disease (4, 5), and amyotrophic lateral sclerosis (6). P7C3 derivatives exert an antidepressant effect in mice by increasing hippocampal neurogenesis (7), and blocking axonal degeneration and perverse function after traumatic brain injury (8), and suppressing neuroinflammation (9). Since P7C3 could enhance the activity of the purified nicotinamide phosphoribosyltransferase (NAMPT) enzyme and increase nicotinamide adenine dinucleotide levels through its NAMPT-mediated salvage, it was confirmed that P7C3 is also an NAMPT activator (10). Although the existing studies have confirmed that P7C3 exhibits a pleasant effect on the neurodegenerative disease and repair of nerve damage, the primary proteins which directly bind to and are modulated by P7C3 in human cells are still poorly known, and there are still no reports about P7C3 having anti-cancer effects.

In our study, we performed a systematic screening of the one-to-one P7C3 interacting proteins by using a human proteome microarray containing 23136 human purified proteins. We identified 577 candidate P7C3 targeting proteins. We found that P7C3 targeting human proteins is mainly involved in energy metabolic pathways, including central carbon metabolism, glutathione metabolism, pyruvate metabolism, and, most importantly, glycolysis, which is one of the most important hallmarks of cancer cells (11, 12). We demonstrated that P7C3 interferes with the normal energy metabolic process in glioma cells by regulating aerobic glycolysis. Regarding the underlying mechanism, we confirmed that P7C3 could directly target phosphoglycerate kinase 1 (PGK1) at several lysine residues and asparagine residues. This P7C3-PGK1 interaction accelerated the PGK1 protein degradation and reduced the protein level and total intracellular kinase activity of PGK1 in glioma cells. PGK1 is a crucial catalytic enzyme in glycolysis and is overexpressed in many cancers (13). An abnormally high level of PGK1 is associated with poor prognosis in patients with glioma. The use of aerobic glycolysis to supply energy for rapid malignant growth is a common feature of most cancer cells (12). Finally, we found that the neurogenic compound P7C3 could significantly inhibit the malignant growth of glioma cells *in vitro* and *in vivo*, while it did not damage normal neural cells at a concentration that exhibited an inhibitory effect on glioma. Taken together, this study is the first to report that the neurogenic compound P7C3 exhibits an inhibitory effect on malignant growth of glioma *in vitro* and *in vivo*, possibly due to its effect on the glycolytic pathway, specifically on PGK1, as the direct target of P7C3 in glioma cells. Most importantly, we were first to identify the target human proteins of P7C3. We believe that these findings may provide important scientific clues for future studies.

## Materials and Methods

### Chemicals, Reagents, and Antibodies

P7C3 (C_21_H_18_Br_2_N_2_O) was purchased from MedChemExpress (HY-15976, Monmouth Junction, NJ, USA). For *in vitro* experiments, P7C3 was dissolved in dimethyl sulfoxide (DMSO) (D8418, Sigma, USA) as a stock solution to obtain a concentration of 20 mM. For *in vivo* experiments, DMSO was used as a co-solvent to aid in the dissolution of P7C3 in normal saline (20 mg/mL), and a dose of 15 mg/kg body was used. Biotin (C_10_H_16_N_2_O_3_S) was purchased from MedChemExpress (HY-B0511, Monmouth Junction, NJ, USA). P7C3-Biotin (P7C3-Bio) was synthesized by Chemdow Biotechnology (Beijing, China). Cy3-Streptavidin was purchased from Sigma-Aldrich (S6402, Sigma, USA). Streptavidin Mag Sepharose™ was purchased from Sigma-Aldrich (GE28-9857-38, Cytiva, USA). Recombinant human PGK1 (GST-PGK1, ag12119, Proteintech, USA) was purchased from Proteinteck Group, Inc. Antibodies used in this study are as follows: HK1 (#2024, CST, USA), HK2 (#2867, CST, USA), GCK (#3782, CST, USA), ALDOA (#8060, CST, USA), PGK1(sc-130335, Santa Cruz, USA), PGK2 (ab183031, Abcam, USA), GST (#2624, CST, USA), GAPDH (#5174, CST, USA), Beclin-1 (#3495, CST, USA), LC3A/B (#12741, CST, USA), Iba1 (ab178846, Abcam, USA), SOX10 (ab227680, Abcam, USA), GFAP (ab68428, Abcam, USA), and β-Tubulin (#2128, CST, USA).

### Databases

The Human Protein Atlas (https://www.proteinatlas.org/) was used to analyze PGK1 and PGK2 mRNA levels in human cell lines. Transcriptomics datasets from TCGA (The Cancer Genome Atlas, https://www.cancer.gov/about-nci/organization/ccg/research/structural-genomics/tcga) and Chinese Glioma Genome Atlas (CGGA, http://www.cgga.org.cn/) were used for analyzing the profile of PGK1 and PGK2 mRNA level in patients with glioma. The data shown in this study, based on the above genome atlases, were downloaded from the Gliovis data portal for visualization and analysis of brain tumor expression datasets: (http://gliovis.bioinfo.cnio.es/) (14).

### Cells and Cell Culture

Human glioma cell lines, U87MG, U251G, and T98G were purchased from the Chinese Academy of Sciences cell bank (Shanghai, China), U118MG were purchased from Obio Technology Co., LTD (Shanghai, China). Glioma cell lines were culture in Dulbecco’s Modified Eagle Medium (DMEM) (17619004, Corning, USA), containing 10% fetal bovine serum (10099141C, Gibco, USA), incubated at 37°C in a humidified atmosphere containing 5% CO_2_. Normal human astrocytes (HA) were purchased from Cell Science and cultured in astrocyte medium (#1801, ScienCell, USA) incubated at 37°C humidified atmosphere containing 5% CO_2_.

### Proteome Microarrays Assay and Data Processing

Identification of P7C3 targeting proteins was performed by Wayen Biotechnology (Shanghai, China) using a HuProt™ 20K Proteome microarray containing 23,136 purified human proteins. Firstly, blocking buffer (1% bovine serum albumin in 0.1% Tween 20) was used to block the microarrays at room temperature (RT) for 1 h. Then, the microarrays were incubated with 10 μM P7C3-Bio or 10 μM biotin at RT for 1 h. Next, the microarrays were washed with TBST three times and incubated with Cy3-Streptavidin (diluted in TBST, 1:1000) at RT for 1 h. Finally, the microarrays were washed again and spun to dry. Observations and data analysis were performed by GenePix 4000 microarray scanner (Molecular Devices, USA) and GenePix Pro-6.0 software (Molecular Devices, USA). The protein spot with the signal strength indicator *Z*-Score > 2.8, in a P7C3-Bio-treated microarray, and < 2.8, in a biotin-treated microarray, was identified as the candidate positive protein. The *Z*-Score ratio between P7C3-Bio treated microarray and biotin treated microarray was defined as IMean_Ratio, and the candidate positive protein with IMean-Ratio>1.4 was identified as the final P7C3 target protein.

The enrichment analysis, including the Kyoto Encyclopedia of Genes and Genomes (KEGG) pathway, biological process, and cellular component, was performed using DAVIA 6.7 (15). Protein interaction networks were built automatically using the Search Tool for the Retrieval of Interacting Genes/Proteins (STRING) system (http://string-db.org/). The Network of protein-protein interaction was built by Cytoscape v2.8.1 (http://www.cytoscape.org). Densely connected regions were calculated by using a graph theoretic clustering algorithm called molecular complex detection (MCODE) (16). Motif enrichment analysis was performed by using MEME (version 4.8.1) (17).

### Oxygen Consumption Rate (OCR) and Extracellular Acidification Rate (ECAR) Assay

Seahorse XF96 Extracellular Flux Analyzer was used for measuring cell oxygen consumption rate (OCR) and extracellular acidification rate (ECAR). U87MG or U118MG cells were plated in XF96 cell culture plates (Seahorse Bioscience, USA), and incubated for 8 h at a normal cell incubator. Then, cells were treated with a new medium containing 0.3% DMSO (0 μM P7C3), 30 μM P7C3, or 50 μM P7C3 for 24 hours. Then, cells were equilibrated with bicarbonate-free buffered DMEM for 1 h without CO2 immediately before the extracellular flux (XF) assay. The glycolytic rate assay was performed in XF Base Media without phenol red, containing 5 mM HEPES, 10 mM glucose, 1 mM sodium pyruvate, and 2 mM L-glutamine, and rotenone/antimycin A (0.5 μM) and 2-deoxy-glycose (50 mM) were added in proper order. The mitochondrial stress test was performed in XF Base Media containing 10 mM glucose, 1 mM sodium pyruvate, and 2 mM L-glutamine, and oligomycin (1 μM), carbonyl cyanide 4-(trifluoromethoxy) phenylhydrazone (1 μM), and rotenone/antimycin A (0.5 μM) were added in proper order.

### Glucose Uptake Assay

Glucose Uptake Assay Kit (#KA4086, Abnova, Taiwan) was used for glucose uptake measuring. First, 5 × 10^4^ cells/well/100 μL/were seeded in a 96-well plate in treatment medium which containing 0.3% DMSO (0 μM P7C3), 30 μM P7C3, or 50 μM P7C3 for 18 h. The medium was then replaced with a new growth medium, and the cells were cultured for another 6 hours before experiments. Then, the cells were treated as desired, and 10 μL/well 2-Deoxy-d-glucose was added, and the cells were incubated at 37°C for 30 min. To wash and lyse cells, 50 μL/well 2-Deoxy-d-glucose uptake assay working solution was added, and the cells were incubated at RT for 90 min. The optical density (OD) ratio at 570/610 nm was measured using a spectrophotometer (1510, Thermo Fisher, USA).

### Measurement of Intracellular Pyruvate, Lactate, and ATP Levels

Pyruvate assay kit (BC2205, Solarbio, China), lactate assay kit (BC2235, Solarbio, China), and ATP assay kit (BC0300, Solarbio, China) were used to measure the intracellular levels of pyruvate, lactate, and ATP, respectively. Glioma cells were treated with 0.3% DMSO, 30 μM P7C3, or 50 μM P7C3 for 24 hours. After that, an equal number of cells (5 × 10^6^ cells from each sample) were collected for the subsequent measuring procedure according to the product manual. Relative absorbance was measured under a spectrophotometer (1510, Thermo Fisher, USA).

### Measurement of Total Intracellular Kinase Activity of PGK1

The total intracellular kinase activity of PGK1 in glioma cells was measured using phosphoglycerate kinase activity assay kit (ab252890, Abcam, USA) according to the manufacture’s protocol. Briefly, cells were treated with P7C3 for 24 h, and 4 × 10^6^ cells from each sample were harvested and treated with cold PGK assay buffer for 10 min on ice. Then, the samples were centrifuged at 10 000 g for 5 mins, and the supernatant was collected. An Ammonium sulfate solution was used for precipitation. Samples were centrifuged at 10 000 g at 4°C for 10 mins, and the PGK assay buffer was added to resuspend the pellet. Each sample (50 μL) of each sample was mixed with 50 μL of reaction mix buffer, and the absorbance was measured immediately at OD340 at two points, with an interval of 30 minutes. The OD340 difference between the two time points was used to calculate the PGK1 activity.

### Intracranial Glioma Xenograft Model

U87MG-Luc cells used in the intracranial glioma xenograft model were purchased from Obio Technology Co., LTD (Shanghai, China). Briefly, 3 × 10^5^ cells were stereotactically injected into 6-weeks-old male BALB/cA nude mice (HFK BIOSCIENCE, China) (12 mice per group). Transplanted cells *in vivo* grow for 7 days. Then, the mice were divided into the control group and treatment group based on tumor size. The mice in the control group were given daily intraperitoneal injections of normal saline, while the treatment group was given a daily intraperitoneal injection of 15 mg/kg/body P7C3. On day 14 after beginning P7C3 treatment, tumor size *in vivo* was measured again. Tumor size was measured using an IVIS imaging system (IVIS Lumina II, USA). The photon flux was used to evaluate the tumor size. Subsequently, three mice from each group were used to obtain 4% PFA perfusion and paraffin section, and three mice in each group were used for collecting the total protein of xenograft tumor. The remaining six mice in each group were used for Kaplan–Meier survival analysis of the tumor-bearing mice. Animal experiments in this study were performed in accordance with the relevant guidelines and were approved by the Ethics Committee of the Southern Medical University and The Seventh Medical Center of General Hospital of PLA.

### Immunohistochemistry Analysis

Tumor tissues, obtained from the intracranial glioma xenograft model on day 21 after U87MG cells transplantation, were used for immunohistochemistry staining assay. The slide was dried at 60°C for 1 h, deparaffinized with xylene, and rehydrated with gradient alcohol. Tris-EDTA buffer was used for antigen retrieval, and a 3% H_2_O_2_ solution was used to eliminate endogenous peroxidase activity. The slides were then incubated with the primary antibodies at 4°C overnight. The antibodies were detected with Polink-2 plus polymer horseradish peroxidase detection system (ZSGB Bio, China). Images including tumors and surrounding mouse brain tissue were taken under a Leica microscope (DM2000B, Germany). Images were evaluated using Image-Pro Plus software (Media Cybernetics, USA).

### TUNEL Assay

The in-situ cell death detection kit-POD (11684817910, Roche, Switzerland) (TUNEL) was used for detecting apoptotic cells in tumor tissues obtained from the intracranial glioma xenograft model. The slides were dried at 60°C for 1 h, deparaffinized with xylene, and rehydrated with gradient alcohol. Tris-EDTA buffer was used to conduct antigen retrieval and 3%-H_2_O_2_ solution was used to eliminate endogenous peroxidase activity. The slides were then incubated with a terminal deoxynucleotidl transferase reaction mix for 1 h at 37°C. Next, the slides were washed with phosphate-buffered saline (PBS), and incubate with streptavidin-biotin-peroxidase for 30 min, stained with 3,3′-diaminobenzidine tetrahydrochloride, and counterstain slides with hematoxylin. Slides were observed and photographed under a Leica microscope (DM2000B, Germany).

### Cell Viability Assay

The effect of P7C3 on cell viability was analyzed using Enhanced Cell Counting Kit-8 (CCK-8, C0041, Beyotime, China). Briefly, 1 × 10^4^ cells were seeded in a 96-well plate and culture in a growth medium for 8 h. The medium was replaced with a treating medium containing P7C3 at concentrations of 0, 10, 20, 30, 40, 50, 75, 100, 150, and 200 μM. The cells were then cultured for another 24 h. Next, the medium was removed, and Cell Counting Kit-8 reagent was added, and the cells were further incubated at 37°C for 1 h. Finally, the OD450 value was measured using a spectrophotometer (1510, Thermo Fisher Scientific, USA).

### Apoptosis Assay

An Annexin V-FITC Apoptosis Detection Kit (C1062L, Beyotime, China) was used for apoptosis assay. Glioma cells and normal human astrocytes were treated with P7C3 at a concentration of 0 μM or 35 μM (near half-maximal inhibitory concentration [IC50] of U87MG or U118MG) for 24 h. Then, cells were harvested and washed by PBS buffer. Cells (5 × 10^4^) cells collected and resuspended in 195 μL Annexin V-FITC binding buffer. Next, 5 μL Annexin V-FITC and 10 μL PI were added to the cells, mixed well, and the mixture incubated at RT for 20 mins away from light. The results were determined using flow cytometry (BD Accuri™ C6 Plus Flow Cytometer, BD, USA).

### EdU Incorporation Assay

BeyoClick EdU Cell Proliferation Kit with Alexa Fluor 555 (C0075L, Beyotime, China) was used for the EdU incorporation assay. Briefly, cells were culture in a 12-well plate overnight, and then P7C3 was added at a final concentration of 35 μM for 24 h. Next, the medium was replaced with 20 μM EdU solution, and the cells were further incubated for 2 h at 37°C. Subsequently, 4% paraformaldehyde solution was used to fix cells for 10 min, followed by permeabilization of the cells for 15 min at RT using 0.5% Triton X-100 solution. The cells were washed three times with PBS and then incubated with click reaction solution for 30 minutes at RT away from light. Finally, the cells were rinsed and stained with 4′,6-diamidino-2-phenylindole. The results were analyzed using a Leica DMI3000B fluorescence microscope (Leica, Germany).

### Cell Invasion Assay

The transwell system (CLS3379, Corning, USA) was used for the cell invasion assay. The wells were firstly coated with 12.5% Matrigel (1:8 in DMEM, BD354248, BD Biosciences, USA) at 37°C for 1 h, and then rinsed with DMEM before use. Then, 1 × 10^5^ cells in 200 μL DMEM containing 1% fetal bovine serum and 0 μM or 35 μM P7C3 were seeded in the upper chamber, and 600 μL DMEM which containing 20% fetal bovine serum and 0 μM or 35 μM P7C3 was added to the lower chamber. Cells were cultured in the chamber for 24 h. Then, the medium was removed, the cells were fixed with methanol, and the cells were stained with 0.1% crystal violet. Invasion cells were photographed and counted for statistical analysis.

### Wound-Healing Assay

Cells (5 × 10^5^) were seeded in 6-well plates at 90% confluency. Scratches of a predetermined length were vertically introduced into the monolayer using a 200 μL pipette tip. Then, the plate was rinsed to remove the detached cells. Next, a culture medium containing 0 μM or 35 μM P7C3, and 5 mM hydroxyurea (H8627, Sigma, USA) for distinguishing the contributions of cell proliferation to wound closure from those of migration, was added. The wounds were photographed first, and the 6-well plate was placed back into the incubator for cultivation for 16 h. Then, the wounds were photographed again. Migration cells were counted for statistical analysis.

### Quantitative Real-Time PCR (qRT-PCR) Assay

*PGK1* mRNA levels were measured using a quantitative-PCR assay. Cells were treated with 0 μM or 30 μM P7C3 for 24 h. Total RNA was extracted by a TRIzol Plus RNA Purification Kit (12183555, Invitrogen, USA) according to the manufacturer’s protocol. PrimeScript RT Reagent Kit (RR037B, TaKaRa, Japan) was used to construct cDNA. qRT-PCR analysis was performed by Platinum SYBR Green qPCR SuperMix-UDG w/ROX kit (11744100, Thermo, USA) according to the protocol. Quantitative PCR reactions were repeated at least three times. The expression level was normalized to *β-actin*. The primer sequence information was as follows: for *PGK1* the forwards primer is 5’- ATCTGCCACAGAAGGCTGGT-3’, and the reverse primer is 5’- ACTTTAGCTCCGCCCAGGAT-3’; for *β-actin*, the forwards primer is 5’- GGCACCCAGCACAATGAAGA-3’, and the reverse primer is 5’- CATTTGCGGTGGACGATGGA-3’.

### Western Blot

Total protein in cells was extracted with radioimmunoprecipitation assay lysis buffer (P0013B, Beyotime, China) supplemented with protease inhibitor (P1011, Beyotime, China). Protein concentration was measured using a bicinchoninic acid protein assay kit (PC0020, Solarbio, China). Proteins were separated by SDS-PAGE and transferred to a polyvinylidene fluoride membrane. QuickBlock™ Blocking Buffer for Western Blot (P0252, Beyotime, China) was used for antigen blocking at RT for 15 min. Then, the membranes were incubated with primary antibodies (1:1 000 diluted in QuickBlock™ Blocking Buffer) overnight at 4°C. Membranes were then washed thrice with cool TBST Buffer (ST673, Beyotime, China), followed by incubation in horseradish peroxidase-linked antibody (#7076 & #7074, CST, USA) at RT for 1 h. Membranes were washed thrice with cooled TBST Buffer thrice and then reacted with Immobilon Western Chemiluminescent horseradish peroxidase Substrate (P90719, Millipore, USA) for 10 s. The protein bands in the membrane were detected using ChemiDoc™ XRS+ with Image Lab™ Software (721BR05475, Bio-Rad, USA). Quantification analysis of the related protein was performed densitometrically using ImageJ software.

### Streptavidin Agarose Affinity Assay

To identify P7C3 binding to the intracellular target protein of glioma cells, two experimental methods were adopted. Firstly, glioma cells cultured in the dish were first treated with 50 μM P7C3-Bio for 24 h, and then the cell lysates were extracted using radioimmunoprecipitation assay buffer. Secondly, cell lysates extracted from normally cultured glioma cells using radioimmunoprecipitation assay buffer, and then incubated with 10 μM P7C3-Bio at 37°C for 1 h. Subsequently, a mixture based on these two methods was incubated with streptavidin agarose overnight at 4°C. To detect P7C3 binding to purified PGK1 protein, 25 μg recombinant human PGK1 solution was incubated with 10 μM P7C3-Bio at 37°C for 1 h, and then incubated with streptavidin agarose overnight at 4°C. Next, the streptavidin agarose was washed thrice with cool PBS buffer and boiled for subsequent western blot analysis.

### Transmission Electron Microscopy

Glioma cells were treated with P7C3 for 24 h. The culture medium was removed, and 2.5% glutaraldehyde solution (P1126, Solarbio, China) was added to fix the cells for 1 h at RT. Then, cells were harvested, washed gently three times in PBS, and postfixed in 1% osmium tetroxide-PBS for another 1 h. After dehydration in graded ethanol series, cells were critical point dried and sputter-coated with 10% gold using JEOL JFD-320 Cold Ice Dryer and JFC-1600 Ion Sputterer. Observations were performed using a transmission electron microscope (H-7650, Hitachi, Japan) at an accelerating voltage of 80 kV.

### Electrospray Ionization-Liquid Chromatography-Mass Spectrometry (ESI-LC-MSMS) and Data Processing

The P7C3 binding to protein was analyzed using a Q Exactive™ Hybrid Quadrupole-Orbitrap™ Mass Spectrometer (ESI-LC-MSMS, Thermo Fisher Scientific, USA). Briefly, P7C3 was dissolved at a final concentration of 20 μmol/L and incubated with 1 μg/μL recombinant human PGK1 at 37°C for 1 h. Samples were loaded to SDS-PAGE, and the protein bands were excised from SDS-PAGE gel. After digestion, analysis was performed using a mass spectrometer. The raw MS files were analyzed and searched against a protein database based on the species of the samples using Byonic. The parameters were set as follows: the protein modifications were carbamidomethylation (C) (fixed), oxidation (M) (variable), P7C3 (+456.179) (R, N, Q, K) (variable); the enzyme specificity was set to trypsin or chymotrypsin; the maximum missed cleavages were set to 3; the precursor ion mass tolerance was set to 20 ppm, and MS/MS tolerance was 0.02 Da. Only high confident identified peptides were chosen for downstream protein identification analysis.

### Statistical Analysis

GraphPad Prism 8 (GraphPad Software Inc., USA) was used to perform the statistical analysis and statistical chart making. Quantitative data were analyzed by unpaired *t* test, Kaplan–Meier survival analysis with a log-rank test. Each group of experiments was repeated at least three times, standard deviation (SD) was calculated to indicate the variation within each experiment and data, and values represent mean ± SD. *P* < 0.05 is considered to be statistically significant.

## Results

### P7C3 Target Proteins Are Closely Related to Energy Metabolism

**Figure 1:**
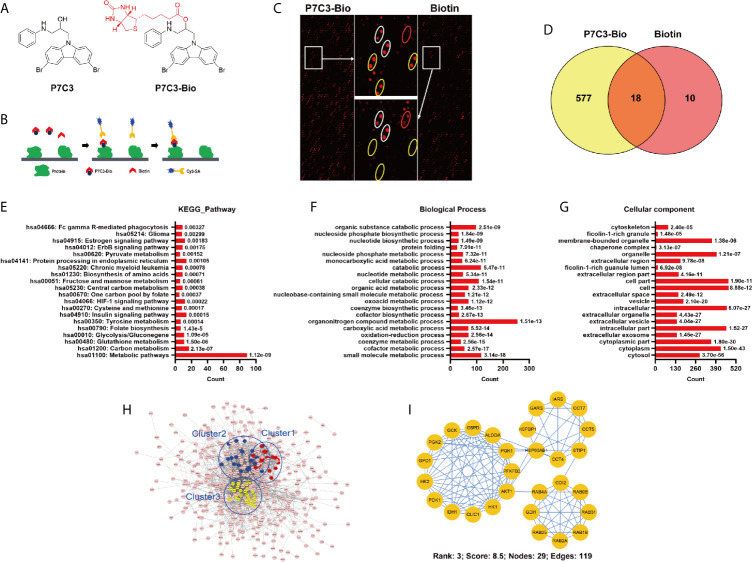
Global profile of P7C3-binding proteins on the human proteome microarrays. **(A)** Molecular structure of P7C3 and biotin-labeled (red) P7C3 (P7C3-Bio). **(B)** The schematic representation of identifying P7C3 target proteins using human proteome microarrays. **(C)** Representative images of protein array showing positive spots (inside the yellow circle) and negative spots (inside the white circle and red circle) on the partial area of microarrays. **(D)** Venn map of the positive spots for P7C3 target proteins and biotin target proteins. The number in the yellow area represents the number of P7C3 specific target proteins. **(E)** Top 20 enriched pathways of P7C3 target proteins. **(F)** Top 20 enriched cellular components of P7C3 target proteins. **(G)** Top 20 enriched biological processes of P7C3 target proteins. **(H)** The complete interaction network of the P7C3 target proteins, the top 3 tightly connected clusters are color-coded. **(I)** The third-ranked cluster is glycolysis, with a score of 8.5.

To comprehensively understand the pharmacological function of P7C3, we explored the P7C3 target proteins using a HuProt™ human protein microarray. We firstly synthesis biotin-labeled P7C3, the molecular structure of which is shown in **Figure 1A**. P7C3-Bio and biotin were used to react with recombinant proteins constructed on a HuProt™ human protein microarray. Proteins bound to P7C3-Bio or biotin were identified by Cy3-conjugated streptavidin (**Figure 1B**). Representative positive and negative target proteins in some regions of the microarrays are shown in **Figure 1C**. The spot inside the yellow circle was the protein that could be directly bound by P7C3-Bio only; the spot inside the white circle was the protein that could be directly bound by P7C3-Bio and biotin together; and the spot inside the red circle was the protein that could be directly bound by biotin only. The spot inside the yellow circle was classified as a positive spot. In this way, we identified 577 candidate P7C3 target proteins (**Figure 1D**).

To thoroughly understand the biological function of P7C3 target proteins, enrichment analysis was performed, including Kyoto Encyclopedia of Genes and Genomes pathway, cellular component, and biological process. The top 20 enriched terms are listed. The top 20 enriched pathways were mainly involved in energy metabolism, including carbon metabolism, glutathione metabolism, glycolysis/gluconegene, central carbon metabolism, and pyruvate metabolism (**Figure 1E**). Glycolysis and pyruvate metabolism are negligible in many cancers and promote the malignant progression of cancers (11, 12, 18). For the cellular component, the top 20 terms suggested P7C3 target proteins are mostly localized in the cytosol (**Figure 1F**). For the biological process, the top 20 terms also indicate that P7C3 target proteins were mainly involved in metabolic processes (**Figure 1G**). In addition, we performed biological interaction networks for P7C3 target proteins to identify significant connections among these P7C3 target proteins (**Figure 1H**). The densest cluster comprised many crucial enzymes, and the top-ranked network cluster 3 was related to glycolytic process regulation (**Figure 1I**). Taken together, P7C3 target proteins are closely related to energy metabolism in human cells.

### P7C3 Regulates Aerobic Glycolysis in Cultured Glioma Cells

Energy metabolism remodeling is an important feature in cancer cells, which is manifested as desirable cooperation between aerobic glycolysis (known as the “Warburg effect”) and tricarboxylic acid cycle (the central route for oxidative phosphorylation), to provide efficient energy for the rapid growth of cancer cells in different tumor microenvironments (19). According to the Warburg effect, cancer cells acquired an increased glucose uptake ability, enabling highly efficient glycolysis and lactic acid fermentation, which, in turn, guarantee a rapid and effective energy supply for maintaining the malignant progress (12, 20). Meanwhile, the tricarboxylic acid cycle is non-negligible for energy production in cancers with deregulated oncogene and tumor suppressor expression, and it also produces metabolites that are crucial for cancer (21, 22). Most cancer cells prefer to produce energy through aerobic glycolysis under a normoxia environment (23).

**Figure 2:**
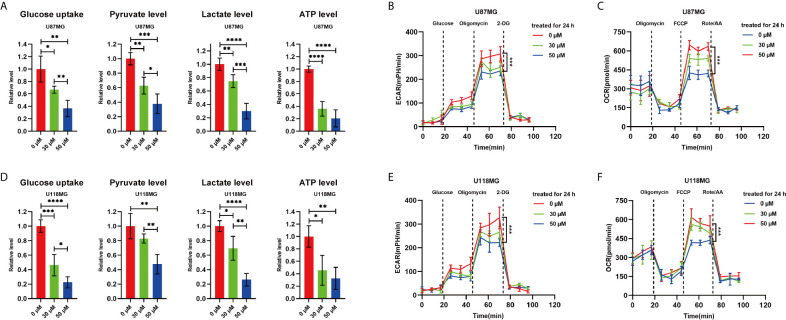
P7C3 regulates aerobic glycolysis. **(A)** Glucose uptake level, pyruvate level, lactate level, and ATP level in P7C3-treated U87MG cells at a concentration of 0 μM, 30 μM, and 50 μM (n = 4). **(B)** Extracellular acidification rate (ECAR) assay using Seahorse XF96 Extracellular Flux Analyzer in P7C3-treated U87MG cells at a concentration of 0 μM, 30 μM, and 50 μM. **(C)** Oxygen consumption rate (OCR) assay using Seahorse XF96 Extracellular Flux Analyzer in P7C3-treated U87MG cells at a concentration of 0 μM, 30 μM, and 50 μM. **(D)** Glucose uptake level, pyruvate level, lactate level, and ATP level in P7C3-treated U118MG cells at a concentration of 0 μM, 30 μM, and 50 μM (n = 4). **(E)** Extracellular acidification rate (ECAR) assay using Seahorse XF96 Extracellular Flux Analyzer in P7C3-treated U118MG cells at a concentration of 0 μM, 30 μM, and 50 μM. **(F)** Oxygen consumption rate (OCR) assay using Seahorse XF96 Extracellular Flux Analyzer in P7C3-treated U118MG cells at a concentration of 0 μM, 30 μM, and 50 μM. (The data were expressed as mean ± SD, *P < 0.05, **P < 0.01, ***P < 0.001, ****P < 0.0001).

To investigate the substantive effect of P7C3 on glycolysis, we measured the ability of glioma cells to consume glucose and a series of metabolites that are involved in glycolysis under normal oxygen conditions cultured cells. The results showed that P7C3 reduced glucose uptake, and the intracellular levels of pyruvate level, lactate level, and ATP in U87MG and U118MG cells (**Figures 2A, D**). Furthermore, P7C3 also significantly down-regulated the extracellular acidification rate (ECAR), which reflects overall glycolytic flux in U87MG and U118MG cells (**Figures 2B, E**). However, P7C3 increased oxygen consumption rate (OCR), which reflects mitochondrial respiration (**Figures 2C, F**). Taken together, these results suggest that P7C3 regulates aerobic glycolysis in cultured glioma cells.

### P7C3 Down-Regulates Protein Level and Total Intracellular Kinase Activity of PGK1 in Glioma Cells

**Figure 3:**
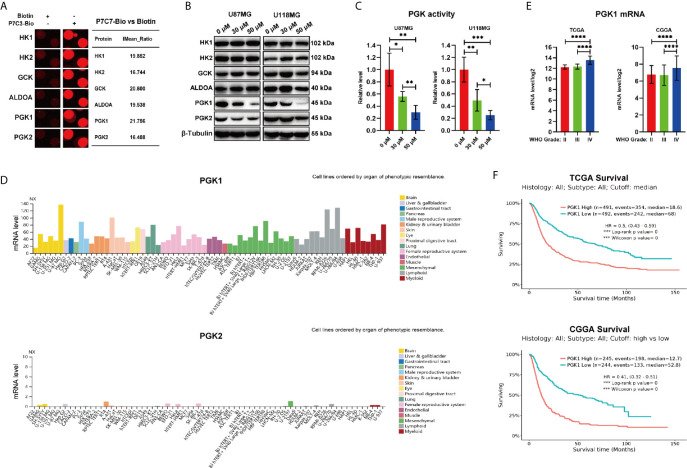
P7C3 reduces the protein level of PGK1 and PGK2. **(A)** Representative spots of P7C3 target proteins, including the glycolytic process. **(B)** Western blotting for detecting protein levels of P7C3 target proteins that are related to glycolysis after U87MG and U118MG cells were treated with P7C3 at a concentration of 0 μM, 30 μM, and 50 μM for 24 h. **(C)** PGK1 activity levels in P7C3-treated U87MG and U118MG cells at a concentration of 0 μM, 30 μM, and 50 μM (n = 4). **(D)** The expression profiles of *PGK1* and *PGK2* mRNA levels. Data were obtained from the Human Protein Atlas database. The images are available at: https://www.proteinatlas.org/ENSG00000102144-PGK1/cell, and (https://www.proteinatlas.rg/ENSG00000170950-PGK2/cell). **(E)** Statistical analysis of *PGK1* mRNA level among WHO grade II-IV glioma. The data were based on TCGA and CGGA and are available at: http://gliovis.bioinfo.cnio.es/. **(F)** Kaplan–Meier survival analysis of *PGK1* mRNA level in glioma patients. The data were based on TCGA and CGGA and are available at: http://gliovis.bioinfo.cnio.es/. (The data were expressed as mean ± SD, *P < 0.05, **P < 0.01, ***P < 0.001, ****P < 0.0001).

Among the 577 candidate P7C3-binding proteins from the microarrays, we focused on GCK, HK1, HK2, PGK1, PGK2, and ALDOA, which are involved in the regulation of the glycolytic process (**Figure 3A**). HK1, HK2, and GCK (HK4) are the members of the hexokinase family which catalyze the conversion of the substrate glucose into glucose-6-phosphate at the first step of glycolysis (24–26). ALDOA is a member of the class I fructose-bisphosphate aldolase protein family and acts as a glycolytic enzyme that catalyzes the conversion of fructose-1, 6-bisphosphate to glyceraldehyde 3-phosphate and dihydroxyacetone phosphate (27). PGK1 and PGK2 are phosphoglycerate kinase family members that catalyze the conversion of 1,3-diphosphoglycerate to 3-phosphoglycerate during the glycolysis process (13, 28). To explore the effect of P7C3 on the expression level of the above proteins, we performed western blot analysis after treating glioma cells with P7C3. The result showed that P7C3 significantly reduced the protein expression level of *PGK1* and *PGK2*, especially *PGK1*, in glioma cells, and the reduction in protein level was concentration-dependent (**Figure 3B**). Next, we detected total intracellular kinase activity of PGK1 after treating glioma cells with P7C3 for 24 h. Results showed that P7C3 clearly down-regulated the total intracellular kinase activity of PGK1 in both U87MG and U118MG cells (**Figure 3C**).

We then analyzed the expression profiles of *PGK1* and *PGK2* in the Human Protein Atlas. We found that among the 69 human cell lines, the mRNA level of *PGK1* was universally high, while those of *PGK2* were low or even absent. Furthermore, among these 69 cell lines, there were three glioma cell lines (U138MG, U251MG, and U87MG), and the mRNA level of *PGK1* was significantly higher than that of *PGK2*. This profile suggested that *PGK1*, but not *PGK2*, is the responsible driver gene in glioma (**Figure 3D**). This result suggested that *PGK1*, but not *PGK2*, potentially plays an important role in promoting glioma. Therefore, we explored the clinical data based on TCGA and CGGA databases to investigate the expression level and the clinical relevance of *PGK1* in the median survival of glioma patients. We found that *PGK1* mRNA levels were higher in high-grade glioma, both in TCGA and CGGA databases (**Figure 3E**). Furthermore, Kaplan–Meier survival analysis of TCGA and CGGA databases showed that patients with glioma and high expression level of *PGK1* exhibited a conspicuously poor overall survival compared with patients with low *PGK1* expression level (**Figure 3F**). Taken together, we found that P7C3 down-regulates the protein level and total intracellular kinase activity of PGK1 in glioma cells.

### P7C3 Directly Targets PGK1

**Figure 4:**
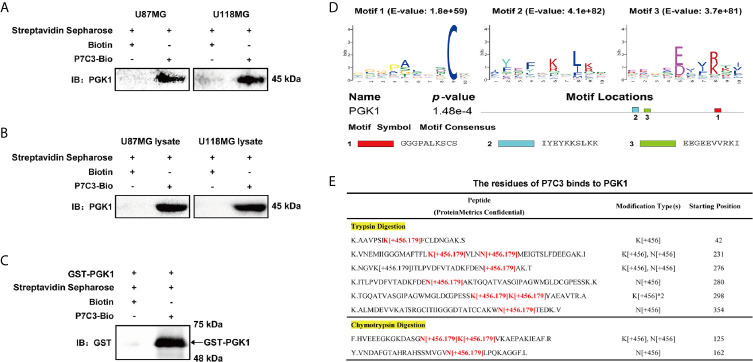
P7C3 directly binds to PGK1. **(A)** Streptavidin affinity assay for P7C3-Bio binds to PGK1 in P7C3-treated glioma cells. Cells in culture were treated with 50 μM P7C3-Bio for 24 h, and then streptavidin agarose was added to incubate with the cell lysates. **(B)** Streptavidin affinity assay for P7C3-Bio binds to PGK1 upon cell lysates. Cell lysates were incubated with 10 μM P7C3-Bio at 37°C for 1 h, and then streptavidin agarose was added to incubate with the cell lysates. **(C)** Streptavidin affinity assay for P7C3-Bio binds to purified PGK1 protein. In total, 25 μg recombinant human PGK1 solution was incubated with 10 μM P7C3-Bio at 37°C for 1 h, and then streptavidin agarose was added to incubate with the cell lysates. **(D)** Motif analysis based on top 100 P7C3 target proteins was performed by MEME, and three motifs were identified. The motif consensus and motif locations at PGK1 are shown. **(E)** The P7C3 binding sites of PGK1 were identified by ESI-LC-MSMS. The P7C3 binding sites were identified by the predicted peptide mass plus P7C3 [C_21_H_18_Br_2_N_2_O with a loss of a water] and minus one H.

To understand the mechanism by which P7C3 reduces the protein level and glycolytic enzyme activity of PGK1 in glioma cells, we first examined the specific interaction between P7C3 and PGK1 using streptavidin affinity assay. We used streptavidin mag sepharose to precipitate P7C3-Bio after treating cells with P7C3-Bio for 24 hours, or after incubating the cell lysate incubated with P7C3-Bio at 37°C for 1 h. In this way, we detected PGK1 by western blotting in both U87MG and U118MG cells (**Figures 4A, B**). Furthermore, the purified recombinant human PGK1 protein (with GST tag) was incubated with P7C3-Bio at 37°C for 1 h, and GST-PGK1 was detected at the corresponding molecular weight position after the precipitation of P7C3-Bio (**Figure 4C**). These results indicate that P7C3 directly binds to PGK1.

To further understand the binding mode between P7C3 and PGK1, we first assessed the consensus sequences among the top 100 P7C3 target proteins by MEME motif assay based on the primary amino acid sequences. We found three consensus motifs, GGGPALKSCS (E-value = 1.8e+59), IYEYKKSLKK (E-value = 4.1e+82), and EEGEEVVRKI (E-value = 3.7e+81) (**Figure 4D**). We then identified the amino acid residues of P7C3 that bind to PGK1. Purified PGK1 was incubated with P7C3 and then used for SDS-PAGE process. Samples were digested by tryptic digestion or chymotrypsin digestion and analyzed by ESI-LC-MSMS. The P7C3-binding sites were identified using the predicted peptide mass plus P7C3. The result showed several mass peaks at many lysine residues (K) and asparagine residues (N) (**Figure 4E**). Combined the results of motif enrichment analysis, we found that lysine is the coincident residue that P7C3 prefers to bind to. Taken together, these findings indicate that P7C3 directly targets PGK1 in glioma cells.

### P7C3-PGK1 Interaction Induces Autophagy-Lysosome-Mediated PGK1 Degradation in Glioma Cells

**Figure 5:**
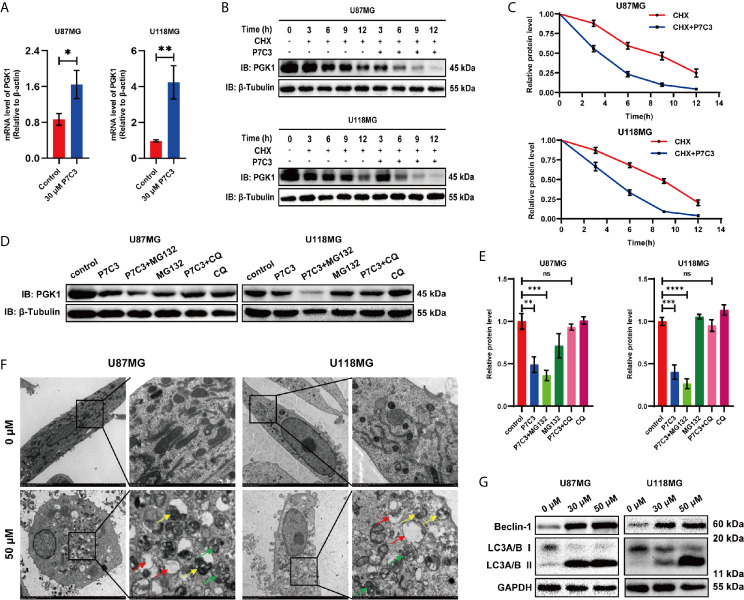
P7C3 accelerates PGK1 protein degradation. **(A)** *PGK1* mRNA level was measured by RT-PCR. P7C3 upregulates the PGK1 mRNA levels in U87MG and U118MG cells (n = 3). **(B)** Time-course assay of P7C3 effects on PGK1 degradation. U87MG and U118MG cells were treated by CHX (100 ug/mL) with or without P7C3 (50 μM) for 24 h, and then PGK1 protein levels were detected by western blotting. **(C)** Protein level curve for PGK1 based on time-course analysis of U87MG and U118MG cells (n = 3). **(D)** Western blotting of PGK1 protein degradation pathway. MG132 (5 μM) and CQ (20 μM) were used to treat cells for 6 h, followed by P7C3 treatment for another 18 h, and the protein level of PGK1 was detected by western blotting. **(E)** The statistical analysis of the quantized protein levels of PGK1 in U87MG and U118MG cells (n = 3). **(F)** Transmission electron microscopy assay after glioma cells were treated with P7C3 for 24 hours. Autophagosomes, autolysosomes, and phagophores are indicated by yellow, green, and red arrows (scale bar: original mage, 5 μm; Zoom image, 1 μm). **(G)** Western blotting for two key autophagy-associated proteins Beclin-1, and LC3A/B, after U87MG and U118MG cells were treated with 0 μM, 30 μM, and 50 μM P7C3 for 24 h. (Data are expressed as mean ± SD, ^ns^ *P* > 0.05, *P < 0.05, **P < 0.01, ***P < 0.001, ****P < 0.0001, ns means none significance).

We initially examined whether the reduced PGK1 protein level caused by P7C3 is dependent on the gene transcriptional level. We measured the *PGK1* mRNA expression by qRT-PCR after glioma cells were treated with P7C3 for 24 h. However, the mRNA level of *PGK1* did not decrease but increased in both U87MG and U118MG cells (**Figure 5A**). This result indicated that the decreased protein levels of PGK1 caused by P7C3 were independent of *PGK1* gene transcription. We then performed a time-course assay of PGK1 degradation. CHX, which is a *de novo* protein synthesis inhibitor, was added to treat the glioma cells, in addition to 50 μM P7C3, and the protein level of PGK1 was detected every 3 h by western blotting (**Figure 5B**). The relative quantification of the PGK1/β-tubulin ratio by densitometry showed that the half-time of PGK1 protein in cells treated with P7C3 and CHX was shorter (about 3.8 h in U87MG cells and 4.4 h in U118MG cells) than that cells treated with CHX alone (about 8.6 h in U87MG cells and 9.0 h in U118MG cells) (**Figure 5C**). These results suggested that P7C3 treatment promoted rapid protein degradation of PGK1.

Next, we used MG132 (a proteasome blocker) and CQ (a lysosome inhibitor) to block the ubiquitin-mediated proteolytic pathway and autophagy-lysosome proteolytic pathway, respectively, and then detected the protein level of PGK1 by western blotting in U87MG and U118MG cells (**Figure 5D**). Quantification of the PGK1/β-tubulin ratio showed that P7C3 significantly decreased the protein levels of PGK1. Furthermore, CQ, but not the MG132, reversed the P7C3-induced reduced protein levels of PGK1 in U87MG and U118MG cells (**Figure 5E**). This result suggested that the accelerated PGK1 degradation caused by P7C3 was dependent on the autophagy-lysosome proteolytic pathway. Then, we performed a transmission electron microscopy assay after treating glioma cells with P7C3 for 24 h. We found the number of autophagosomes, autolysosomes, and phagophorses were significantly increased, indicating that P7C3 activated the autophagy-lysosome proteolytic pathway (**Figure 5F**). In addition, western blotting showed that P7C3 treatment increased the protein levels of Beclin-1 and LC3A/B II, and the ratio of LC3-II/LC3-I was up-regulated, indicating that P7C3 promoted the integrity of autophagy flux in glioma cells (**Figure 5G**). Taken together, these results suggest that P7C3-PGK1 interaction induced autophagy-lysosome-mediated PGK1 degradation in glioma cells.

### P7C3 Inhibits the Malignant Progression of Glioma Cells *In Vitro*

**Figure 6:**
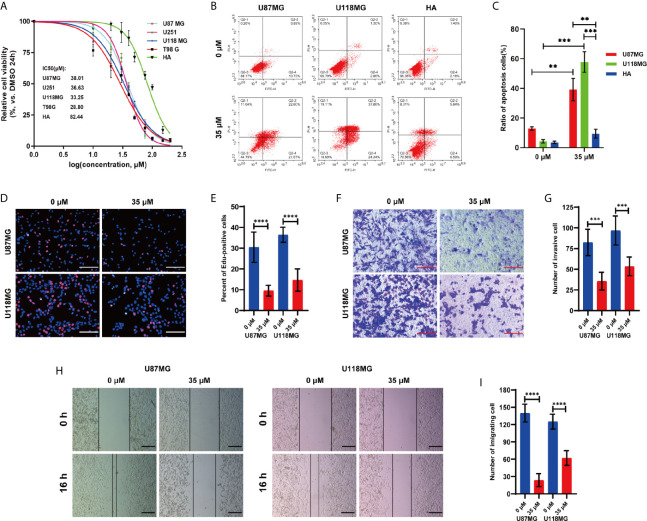
P7C3 inhibits glioma *in vitro*. **(A)** The cell viability curve of different P7C3 concentration effects on four glioma cell lines and human astrocytes (HA) (n = 4). **(B)** Cell apoptosis assay after U87MG, U118MG, and human astrocytes were treated with P7C3 (0 μM and 35 μM) for 24 h (n = 4). **(C)** Statistical analysis of apoptotic cells. P7C3 significantly increased the ratio of apoptotic cells in U87MG and U118MG cells, but the ratio of apoptotic human astrocytes was significantly less (n = 3). **(D)** EdU incorporation assay after U87MG and U118MG cells were treated with P7C3 (0 μM and 35 μM) for 24 h. **(E)** Statistical analysis of the percentage of EdU-positive cells. P7C3 significantly decreased the percentage of EdU-positive U87MG and U118MG cells after P7C3 treatment (n = 12). **(F)** Cell invasion assay after U87MG and U118MG cells were treated with P7C3 (0 μM and 35 μM) for 24 h. **(G)** Statistical analysis of the invasive cells. P7C3 significantly decreased the number of invasive U87MG and U118MG cells after P7C3 treatment (n = 6). **(H)** Wound-healing assay after U87MG and U118MG cells were treated with P7C3 (0 μM and 35 μM) for 16 h. **(I)** Statistical analysis of migrating cells. P7C3 significantly decreased the number of migrating U87MG and U118MG cells after P7C3 treatment (n = 6). (The data were expressed as mean ± SD, **P < 0.01, ***P < 0.001, ****P < 0.0001).

Glycolysis is exacerbated increased in most cancers and has been demonstrated to be a promising target for cancer therapy (18, 29). Since the previous studies have shown that P7C3 could significantly inhibit aerobic glycolysis, we examined whether P7C3 had an anti-cancer effect in glioma. We performed a cell viability assay *in vitro* using four human glioma cell lines and one normal human astrocyte under a drug concentration gradient. The result showed that the IC50 values of P7C3 on glioma cells were mainly within the range between 28.80 μM to 38.01 μM, while the IC50 value of human astrocyte was significantly higher, reaching 82.44 μM (**Figure 6A**). Then, we selected a concentration of 35 μM (IC50 value between U87MG and U118MG) to conduct a flow cytometry assay to detecting apoptosis ratio after P7C3 treatment for 24 h (**Figure 6B**). The statistical result showed that P7C3 induced obvious apoptosis both in both U87MG and U118MG cells but not in human astrocytes at the same concentration (**Figure 6C**).

EdU incorporation analysis was conducted to study the effect of P7C3 on cell proliferation (**Figure 6D**). The statistical result revealed that the percentage of EdU-positive cells was significantly decreased in P7C3 treated U87MG and U118MG cells (**Figure 6E**). We then evaluated the effect of P7C3 on tumor invasion *via* the Transwell assay (**Figure 6F**). The statistical result revealed that the number of invasive cells was significantly reduced in P7C3 treated U87MG and U118MG cells (**Figure 6G**). Finally, we performed a wound-healing assay to evaluate the effect of P7C3 on glioma cells migration, movement, and repair (**Figure 6H**). The statistical result showed that the number of migrating cells was significantly reduced in P7C3 treated U87MG and U118MG cells (**Figure 6I**). The above experiments indicated that P7C3 could inhibit the proliferation, migration, and invasion of glioma cells *in vitro*. Taken together, we found that P7C3 inhibits the malignant progression of glioma cells *in vitro*.

### P7C3 Inhibits the Malignant Growth of Glioma Cells *In Vivo*

**Figure 7:**
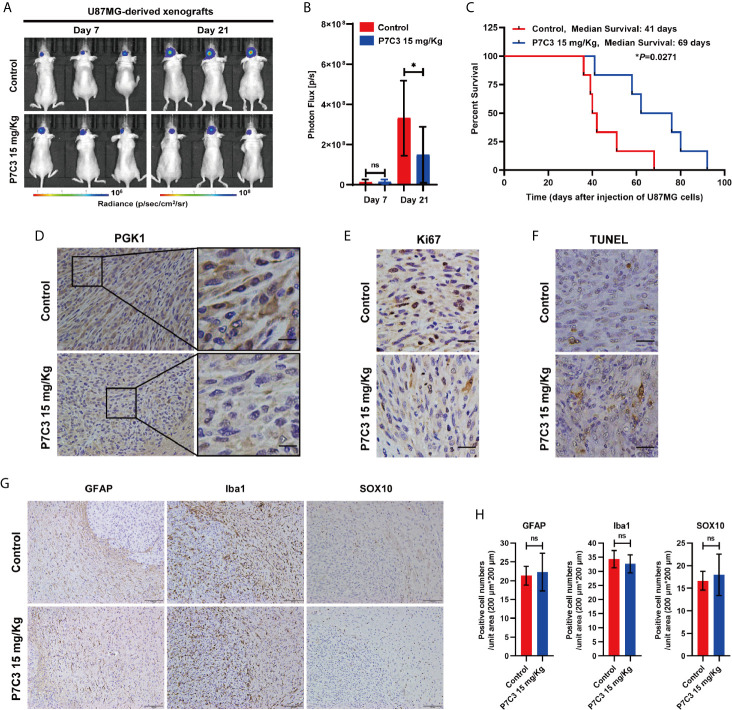
P7C3 inhibits glioma *in vivo*. **(A)** Representative *in vivo* IVIS Luc fluorescence images of xenografts mice on days 7 and 21 after injection of U87MG cells. **(B)** Statistical result of photon flux in xenografts mice on days 7 and 21 (n = 12). **(C)** Kaplan–Meier curves of survival rate of xenografts mice (n = 6). **(D)** Immunohistochemical staining of PGK1 in the indicated tumor tissues was performed, PGK1 positive cells were stained brown (scale bar: 20 μm). **(E)** Immunohistochemical staining of Ki67 in the indicated tumor tissues was performed. Ki67 positive cells were stained brown (scale bar: 50 μm). **(F)** TUNEL analyses of the indicated tumor tissues were performed. Apoptotic cells were stained brown (scale bar: 50 μm). **(G)** Immunohistochemical staining of labeling astrocytes, microglia, and oligodendrocytes in the mice brain by using GFAP, Iba1, and SOX10, respectively (scale bar: 100 μm). **(H)** Statistical analysis of GFAP-positive, Iba1-positive, and SOX10-positive cells in the mouse brain (n = 3). (The data were expressed as mean ± SD, ^ns^ *P* > 0.05, *P < 0.05, ns means none significance).

Next, we assessed the therapeutic effect of P7C3 *in vivo* using intracranial glioma xenograft model. U87MG cells were transplanted into the cerebral of nude mice and allowed to grow for 7 days. Mice were then given daily P7C3 treatment or normal saline treatment for 14 days. Representative *in vivo* IVIS Luc fluorescence images at day 7 and day 21 are shown in **Figure 7A**. The statistical analysis of photon flux in mice suggested that the average intracerebral tumor size was significantly smaller in nude mice treated with P7C3 (**Figure 7B**). In addition, the Kaplan–Meier analysis showed that the median survival time was significantly longer in the nude mice treated with P7C3 (**Figure 7C**). We analyzed the protein expression of PGK1 in the above intracerebral tumor, and the results of immunohistochemistry staining revealed that P7C3 indeed downregulated the protein level in glioma tissue (**Figure 7D**). We also studied the effect of P7C3 on the glioma cell proliferation by Ki67 and cell apoptosis using TUNEL assay *in vivo*. The results showed that P7C3 treatment decreased the number of Ki67-positive cells and increased the TUNEL-positive cells in the intracerebral tumor (**Figures 7E, F**). These results indicate that P7C3 inhibits glioma growth and induces glioma cell apoptosis *in vivo*.

Additionally, we studied the effect of P7C3 on several types of normal glial cells. Immunohistochemical staining was performed to label astrocytes, microglia, and oligodendrocytes using specific markers, GFAP for astrocytes (30), Iba1 for microglia (31), and SOX10 for oligodendrocytes (32), respectively (**Figure 7G**). The statistical results showed that P7C3 did not significantly reduce or increase the number of positive cells in the above-mentioned glia cells (**Figure 7H**). Taken together, P7C3 could inhibit the malignant progression of glioma cells *in vivo*.

## Discussion

Glioma is the most aggressive malignant tumor of the human central nervous system and is characterized by an extremely poor prognosis (33, 34). Since multiple oncogenes or tumor suppressor gene mutations are common, current treatment methods still cannot provide satisfactory therapeutic results (35, 36). In recent years, scientists have been developing more anticancer drugs. In particular, the multi-target anticancer compounds with fewer side effects, are considered promising for future cancer treatment (37). Here, we demonstrated that neurogenic compound P7C3 could inhibit glioma malignant progression *in vitro* and *in vivo* by regulating aerobic glycolysis *via* directly targeting PGK1, which leads to declined energy supply. However, P7C3 does not damage normal glial cells at a concentration that exhibits an inhibitory effect on glioma.

From a pharmacological perspective, the most effective way to elucidate the mechanism of action of a drug compound is to identify the proteins to which it binds. Previous studies have reported that P7C3 is an NAMPT activator (10), while the potential target proteins of P7C3 are still unclear. In this study, we used biotinylated P7C3 to incubate with a human proteome microarray containing 23136 purified human proteins and identified 577 candidate P7C3 target proteins. It is expected that NAMPT is one of the direct target proteins of P7C3, and this may provide evidence that P7C3 enhances NAMPT activity. However, we were surprised to find that the P7C3-binding proteins are mostly involved in cellular signaling pathways of energy metabolism, such as carbon metabolism, glycolysis, amino acid metabolism, and the hypoxia-inducible factor signaling pathway. In addition, pathways of Glioma, chronic myeloid metabolism, and protein processing in the endoplasmic reticulum were also included. Studies have demonstrated that an outstanding feature of cancer is that cancer cells prefer glycolysis to supply advancing energy requirements under normal oxygen conditions, and this feature makes glycolysis feasible for target therapy (18, 19, 23). The results based on proteome microarray implied that P7C3 may be an important agent in regulating glycolysis, which may further affect cancer progression.

Many drugs that directly target glycolytic enzymes have exhibited promising preclinical anti-cancer effects. For example, WZB117 targets GLUT1 (38), lonidamine targets HK2 (39), TLN-232 targets PKM2 (40), and GNE-140 targets LDHA (41). Here, we observed that P7C3 is bound to several key enzymes involved in glycolysis, such as HK1, HK2, GCK, ALDOA, PGK1, and PGK2 in the microarrays. However, we were surprised to find that P7C3 decreased the protein levels of PGK1 and PGK2, especially PGK1. PGK1 is overexpressed in many cancers, and its role in promoting aerobic glycolysis in cancer is associated with poor clinical prognosis (42–44). However, there are few studies on PGK2 expression in tumors. According to TCGA and CGGA databases, only *PGK1* mRNA level is abnormally upregulated in glioma, and the high *PGK1* mRNA levels indicate poor median survival in patients with glioma. However, *PGK2* mRNA levels were very low or even not expressed in gliomas. This result indicates that *PGK1*, but not *PGK2*, is an important promotor of glioma. Our study confirmed that PGK1 is the specific target of P7C3. Most importantly, we identified that P7C3 binds to multiple lysine residues and asparagine residues of PGK1 protein, and this P7C3–PGK1 interaction interferes with PGK1 protein stability and accelerates PGK1 protein degradation through the autophagy-lysosome-mediated pathway. It should be noted that the protein level of PGK1 was reduced, but its mRNA level was increased, as were the mRNA levels of several other glycolysis-related genes, include HK2, PKM2, and TPI (data not shown). Although we did not investigate the underlying mechanism, we confirmed that the declining protein level of PGK1 is not dependent on the mRNA level of PGK1. The posttranslational modification is an important mode of PGK1 activity. For example, the phosphorylation levels of PGK1 at Y324 and T243 are important for its high-efficiency catalytic activity (13, 45), and O-linked N-acetylglucosamine (O-GlcNAc) at T255 could activate PGK1 activity to enhance lactate production (46), and acetylation at K388 could inhibit, while acetylation at K323 could enhance the activity of PGK1 (47, 48). Although the effect of P7C3 on the kinase activity of PGK1 itself has not been determined at present, we demonstrated that P7C3 down-regulates the protein level and total intracellular kinase activity of PGK1 in glioma cells. The results from this study are of great scientific significance as they enhance the possibility of post-translational modification of PGK1 protein, even though we are still unaware of the key residue for P7C3-induced PGK1 degradation.

Since PGK1 is the key glycolytic enzyme in the glycolytic process, we evaluated the metabolic profile in glioma cells after P7C3 treatment and we demonstrated that P7C3 indeed significantly suppressed the aerobic glycolysis in glioma cells, which was manifested as a declining extracellular acidification rate (ECAR), as well as significantly decreasing the intracellular levels of lactate, pyruvate, and ATP. Cancer cells seriously depend on aerobic glycolysis for a cellular process under a normal oxygen environment (11). It is encouraging that our *in vitro* and *in vivo* experiments revealed that P7C3 exhibits an obvious inhibitory effect on malignant growth in glioma cells and this neurogenic compound, and P7C3 does not significantly damage normal glial cells *in vitro* and *in vivo*. These results indicated that P7C3 is a promising anticancer agent for glioma therapy.

In summary, this is the first study to identify direct human protein targets of P7C3, which provides many important scientific clues for the research on the functional mechanism of P7C3 in many fields. We demonstrated that P7C3 is an important regulator of energy metabolism in glioma cells, suggesting that P7C3 regulates aerobic glycolysis by directly targeting PGK1. The underlying mechanism of P7C3 involves direct binding to PGK1 at lysine and asparagine residues, and the specific interaction induces accelerated PGK1 protein degradation through the autophagy-lysosome-mediated pathway. Of note, this is the first study to demonstrate that neurogenic compound P7C3 exhibits an inhibitory effect on malignant growth in glioma and has lesser damaging effects on normal glial cells.

## Data Availability Statement

The datasets presented in this study can be found in online repositories. The names of the repository/repositories and accession number(s) can be found in the article/**Supplementary Material**.

## Ethics Statement

The animal study was reviewed and approved by ethics committee of the Southern Medical University and The Seventh Medical Center of General Hospital of PLA.

## Author Contributions

WC and RX conceived and designed the experiments. WC, WJ, CW, LC, KS, JW, BD, and NL performed the experiments. WC, RX, WJ, and CW collected and analyzed the data. WC and RX wrote the original manuscript. RX reviewed and edited the manuscript. All authors agree to be accountable for the content of this work. All authors contributed to the article and approved the submitted version.

## Funding

This work was supported by a grant from the National Natural Science Foundation of China (Grant No. 81573774), and the Military Medical Science Research Project (16CXZ001).

## Conflict of Interest

The authors declare that the research was conducted in the absence of any commercial or financial relationships that could be construed as a potential conflict of interest.
